# Partial thickness corneal tissue as a patch graft material for prevention of glaucoma drainage device exposure

**DOI:** 10.1186/s12886-016-0196-2

**Published:** 2016-02-27

**Authors:** Oriel Spierer, Michael Waisbourd, Yitzhak Golan, Hadas Newman, Rony Rachmiel

**Affiliations:** Department of Ophthalmology, Tel Aviv Sourasky Medical Center, Sackler Faculty of Medicine, Tel Aviv University, 6 Weizmann Street, Tel Aviv, 64239 Israel; Wills Eye Hospital, Philadelphia, PA USA

**Keywords:** Corneal patch graft, Glaucoma drainage device, DSEK, Conjunctival erosion, Tube exposure

## Abstract

**Background:**

To protect from erosion of the tube in glaucoma drainage device (GDD), the tube is covered by a biologic tissue which is roofed by the conjunctiva. Sclera, pericardium, dura mater and cornea are available as a patch graft. Drawbacks of some of these materials may include high cost and poor appearance. The purpose of this study is to report the long-term outcomes of partial thickness corneal grafts to cover the tube and prevent its exposure, in GDD surgeries.

**Methods:**

This was a retrospective review of all patients who underwent Ahmed glaucoma valve implantation and had a minimum follow-up of 12 months. The tube was covered by a 300-micron partial thickness corneal graft taken either from a previous Descemet stripping endothelial keratoplasty procedure or cut from a whole corneal graft button unsuitable for keratoplasty.

**Results:**

Forty-four patients (45 eyes, mean follow-up of 27.6 ± 11.4 months) were enrolled. The partial thickness corneal grafts maintained clarity throughout follow-up with satisfactory cosmetic results. Mild conjunctival retraction occurred in 4 eyes (8.9 %) between 1 and 12 months after the surgery. Corneal graft melting occurred in 3 (6.7 %) eyes. Tube exposure and additional surgery to re-patch or suture the conjunctiva over the tube was needed in 1 (2.2 %) eye. None of the patients had graft infection or immunologic rejection.

**Conclusions:**

Partial thickness corneal grafts have favorable long-term outcome as a patch for GDD tubes with low rates of tube exposure and other complications.

## Background

Glaucoma drainage device (GDD) surgeries are traditionally performed after failed trabeculectomy or in cases where trabeculectomy is at high risk for failure, such as in the management of complex glaucomas [1]. The more commonly used GDDs in current practice are the Ahmed, Baerveldt and Molteno implants [1]. A GDD is composed of a plate and a tube running along the sclera into the anterior chamber. To protect it from erosion, the tube is covered by a biologic tissue which is roofed by the conjunctiva. A GDD is, however, a foreign device that is implanted onto and into the eye and therefore bears surgical complications associated with foreign objects, among them conjunctival erosion that results in plate or tube exposure, diplopia and inflammation [2]. Covering the tube with a patch graft is intended to prevent the conjunctival erosion and the consequent tube exposure that put the eye under the risk of late endophthalmitis [3]. Sclera, pericardium, dura mater and cornea are available as a patch graft [4], but there are few reports that compare them [5, 6]. The use of a patch graft has some drawbacks, such as cost [7] and the fact that it is a foreign body with the potential to erode the overlying conjunctiva [7]. Specifically, the disadvantage of the scleral patch graft is its thickness [5], which may be a cosmetic issue. On the other hand, a pericardium patch might be too thin and a dura matter patch is expensive [5]. These shortcomings have led some investigators to seek other solutions which do not use patch grafts in GDD surgeries, but rather use a scleral tunnel for the tube [7, 8]. Using partial thickness corneal patch graft may resolve some of the above concerns. The purpose of this study was to report the long-term outcomes and complications of employing a partial thickness corneal graft to cover an Ahmed glaucoma valve implant.

## Methods

### Data collection

Demographic and perioperative data were collected through a retrospective record review of all patients who underwent GDD surgery with partial thickness corneal graft covering between 2007 and 2011 at the Tel Aviv Sourasky Medical Center. Inclusion criteria were age above 18 years at the time of surgery and a minimum follow-up of 12 months. Patients were excluded if they had undergone a previous GDD surgery or if they had a past or current ocular surface disease that could affect the healing of the covering conjunctiva. Patients who had concomitant surgeries, such as pars plana vitrectomy or cataract surgery, were also excluded.

Corneas were obtained from the Tel Aviv Sourasky Medical Center Lions Eye Bank after being approved for transplantation. Corneas were stored in Eusol-C Corneal Storage Media (AL.CHI.MI.A.SRL, Padova, Italy). Tissue evaluation included serology for hepatitis B and C, as well as for HIV. All surgeries were performed by one surgeon (RR) who was also responsible for the clinic visits that included intraocular pressure (IOP) measurements with Goldmann applanation tonometry (AT 900, Haag-Streit AG, Koeniz, Switzerland) before and periodically after surgery. The study followed the tenets of the Declaration of Helsinki and was approved by the ethics committee of the Tel-Aviv Sourasky Medical Center.

### Surgical technique

All GDDs were implanted through a fornix-based incision using the FP7 Ahmed glaucoma valve (New World Medical, Inc., Rancho Cucamonga, CA), with the plate secured 8–12 mm from the corneoscleral limbus. Thirty-three eyes (73.3 %) underwent superotemporal GDD implantation, 11 eyes (24.4 %) underwent inferotemporal implantation, and 1 eye (2.2 %) underwent inferonasal implantation.

After priming, the tube was inserted through a scleral track posterior to the limbus by a 23-gauge needle, and introduced by an inserter into the anterior chamber. Viscoelastic was injected into the anterior chamber to maintain its proper depth. The partial thickness corneal graft, which was used to cover the tube, was sutured to the sclera with four 10-0 nylon sutures. The conjunctiva was closed meticulously with 8-0 vicryl and 10-0 nylon sutures, taking care to ensure that it fully covered the corneal graft.

A partial thickness corneal graft was taken either from a previous Descemet stripping endothelial keratoplasty (DSEK) procedure or cut from a whole corneal graft button. Corneal grafts prepared for a DSEK procedure were cut by an automated microkeratome system (Moria ALTK System, Antony, France) with a 300 micron head passed to create a lamellar dissection. This formed an anterior cap mainly composed of stroma and a posterior endothelial lamellar donor graft. The posterior corneal lamella was used for the DSEK procedure, while the anterior corneal lamella was either discarded or used to cover the tube in the GDD surgery of a different patient. In other cases, a partial thickness corneal graft was prepared from a full-thickness corneal graft that was unsuitable for keratoplasty (i.e., donor age >80 years, low graft clarity, endothelial cell count <2000 cells/mm^2^ and expired tissue [7–60 days from harvesting]).

### Statistical analysis

All the data were recorded on Microsoft Excel™ spreadsheets. A paired-samples *t*-test was used to compare mean pre- and postoperative IOPs. Bonferroni correction for multiple comparisons and a mixed model for repeated measures were also used. Analyses were two-tailed, and significance was set at the 5 % level. Statistical analysis was performed with SPSS™ software V.21 (SPSS, Inc., Chicago, IL).

## Results

Forty-four patients (45 eyes), comprised of 25 males and 19 females with a mean postoperative follow-up of 27.6 ± 11.4 months (range 12–60 months) fulfilled the inclusion criteria and were included in the analysis. Their demographic characteristics and clinical data are summarized in Table 1. The glaucoma subtypes of the study population are summarized in Table 2. The postoperative IOP levels at the different follow-ups were consistently lower (*P* < 0.001) than the preoperative IOP values (Table 1). These differences remained significant after Bonferroni correction for multiple comparisons and application of a mixed model for repeated measures. The mean IOP reduction (i.e., the last recorded preoperative IOP compared to the last recorded postoperative IOP) was 50 %. The mean number of topical anti-glaucoma medications decreased from 3.0 ± 0.9 (range 1–4) to 1.3 ± 1.2 (range 0–4) on the last postoperative follow-up.

**Table 1:** Demographic and clinical data of 44 patients (45 eyes) who underwent glaucoma drainage device surgery

Male/female (%)		56.8/43.2
Age (years)		71.0 ± 10.8 (range 34–92)
Preop.duration of glaucoma (months)		116.2 ± 105.8 (range 1–396)
Trabeculectomies before surgery (n)		0.9 ± 0.9 (range 0–4)
Right/left eye (%)		55.6/44.4
Mean intraocular pressure (mmHg)		
	Preoperative	30.1 ± 8.1
	Postop. day 1	10.9 ± 5.4*
	Postop. week 1	10.5 ± 5.2*
	Postop. month 1	12.7 ± 5.8*
	Postop. month 3	14.1 ± 6.4*
	Postop. month 6	13.3 ± 5.3*
	Postop. year 1	13.8 ± 4.9*
	Postop. year 2	12.8 ± 3.6*
	Postop. final visit	14.5 ± 4.9*

*Postop* postoperative

*P < 0.001 (compared to preoperative)

**Table 2:** Subtype of glaucoma in 44 patients (45 eyes) who underwent glaucoma drainage device surgery

Subtype	n (%)
Pseudoexfoliation glaucoma	11 (25.5)
Open angle glaucoma	9 (20.5)
Neovascular glaucoma	9 (20.5)
Post penetrating keratoplasty glaucoma	6 (13.6)
Uveitic glaucoma	4 (9.1)
Chronic angle closure glaucoma	2 (4.5)
Malignant glaucoma	1 (2.3)
Angle recession	1 (2.3)
Normal tension glaucoma	1 (2.3)

Thirty-three (73.3 %) partial thickness corneal grafts were retrieved from previous DSEK procedures and 12 grafts (26.7 %) were prepared during the GDD surgery from full corneal grafts that were unsuitable to serve as an optical corneal graft. The mean corneal patch diameter was 8.3 ± 0.2 mm (range 8.0–.0 mm). The conjunctiva that covered the corneal patch graft had a button hole during surgery in 1 case (2.2 %). The conjunctival edges were approximated by 8-0 vicryl sutures. There were no other intra-operative complications.

None of the patients had graft infection or immunologic rejection and the partial thickness corneal grafts maintained clarity throughout follow-up. There were no GDD-related complications, such as diplopia, inflammatory reaction in the conjunctiva or plate exposure. Four eyes (8.9 %) had conjunctival retraction between 1 and 12 months after the surgery. As the conjunctival retraction was minimal, Seidel test was negative, and the tube was well-covered by the patch graft, these patients were observed without treatment. Conjunctival erosion with tube exposure occurred in 1 case (2.2 %) with superotemporal GDDs. In this patient the corneal patch graft was also melting 1 month after surgery (Table 3). The patient underwent re-patching with another corneal graft and conjunctival closure. Three eyes (6.7 %) had corneal graft melting (Table 3): in one of them it occurred with conjunctival erosion and tube exposure as was described. In the other 2 patients graft melting occurred 12 and 33 months postoperatively. As the overlying conjunctiva was intact they were observed expectantly without the need for surgical intervention.

**Table 3:** Description of 3 patients with partial thickness corneal patch graft melting

Characteristics	Patient 1	Patient 2	Patient 3
Gender	Male	Female	Male
Age (years)	74	66	79
Eye	Left	Right	Left
Type of glaucoma	Pseudo-exfoliation	Uveitic glaucoma	Pseudo-exfoliation
IOP before surgery (mmHg)	25	25	24
Trabeculectomies before surgery (n)	4	1	1
Location of Ahmed valve	Inferotemporal	Inferotemporal	Superotemporal
Medications before/after the surgery (n)	4/4	3/2	4/0
Corneal patch graft diameter (mm)	8.5	8.25	8.5
Complications during surgery	None	None	None
Post-surgery melting (months)	33	12	1
Follow-up duration (months)	42	36	32

*IOP*, intraocular pressure

## Discussion

Partial thickness corneal patch grafts covering GDD tubes have favorable long-term outcomes. According to our results, these grafts are associated with a 6.7 % rate of corneal graft melting and a 2.2 % rate of tube exposure. Only one case necessitated additional surgery to cover the exposed tube. A GDD transports aqueous humor from the eye through a tube to an episcleral plate located in one of the quadrants of the globe. The aqueous diffuses through the capsular wall and is absorbed by periocular lymphatics and capillaries [3, 9]. Post-surgical complications unique to GDDs include tube-endothelial contact which may cause significant corneal endothelial damage, fibrous encapsulation leading to filtration failure, continuing low-grade inflammation, and tube or plate migration or extrusion [2].

Conjunctival retraction leading to patch graft exposure is usually considered a minor complication, as long as the tube is well covered by the patch graft [10]. This condition, which does not require surgical intervention, was previously found in 33.5 % of eyes undergoing GDD implantation [10], compared with 8.9 % in the current study. Nevertheless, if the conjunctival retraction is significant and the tube is exposed, this may place the eye at risk for infection, especially if Seidel test is positive. Repairing the exposure in these cases may be challenging, especially if the conjunctiva is scarred.

Direct contact between the conjunctiva and the tube will cause the tube to erode the overlying conjunctiva over time and become exposed [7], putting the eye at risk of infection and endophthalmitis [3]. The most vulnerable site for tube erosion is at its entrance through the sclera, and the role of the patch graft is to prevent tube erosion [3]. Although this approach has been shown to decrease exposure rates, it does not always prevent this complication [7]. The reported rates for tube exposure or device extrusion range between 3 and 30.5 % [7, 10–13], putting our 2.2 % rate at the bottom of the scale. In the event of tube or plate exposure, the condition of the conjunctiva and patch graft needs to be assessed. The majority of those cases are effectively managed by conjunctival suturing with patch grafting, while poor conjunctiva quality may necessitate the use of a conjunctival auto-graft, amniotic membrane or buccal graft [14, 15]. Re-patching with a different material should be considered in the event that the patch graft has melted [16]. However, removal of the plate or tube may nevertheless be necessary [13] as had been the case in one of our subjects. Causes for tube exposure have not been clearly elucidated in the literature [17], and the reported risk factors are inconsistent. Potential risk might be related to conjunctival status (as affected by aging, inflammation, and device mobility) [18], the number of previous ocular surgeries [18], the number of preoperative hypotensive medications [10], and a previous trabeculectomy [17]. Inferior quadrant implantation bears a higher risk of conjunctival erosion over the plate, possibly because of exposure or a shallower space within the inferior fornix and less conjunctiva and Tenon’s capsule for implant coverage [10, 19, 20]. Another report suggested that tube exposure does not differ between Ahmed, Baerveldt, and Molteno implants [21]. However, since that report included 38 studies with many surgeons using different surgical techniques and different patch grafts, it is difficult to arrive at any firm conclusion from those data. The low rate of complications relevant to the conjunctiva and the partial thickness corneal graft in the current study makes it impossible for us to draw conclusions about the risk factors for graft patch melting and tube exposure. Two out of the 3 cases with melting had the implant placed inferiorly. Also, an immune-mediated process with resultant rapid melting may have contributed to the corneal melting in one of our patients who had underlying uveitis.

Considerations in the choice of patch graft material include biocompatibility, availability, immunologic safety, ease of use, cost and cosmetic appearance [22]. The use of allograft materials, among which the most common are sclera, pericardium, dura mater and cornea, has some disadvantages. Insofar as they are foreign bodies, they have a prima facie potential to erode the overlying conjunctiva [7]. Secondly, they involve considerable cost [7]. The main disadvantage of preserved sclera is its thickness [5], which may be an issue of cosmetic appearance. Sterility and variable quality are other concerns [5]. When an autologous scleral patch graft is used, large areas of sclera may be thinned for harvesting the graft, thus carrying the risk of perforation as well [22]. As for a pericardium graft, it may be a priori too thin and also prone to more melting with longer follow-up [5]. Both pericardium and dura matter are expensive [5]. The use of gamma-irradiated cornea (VisionGraft) for the coverage of a glaucoma tube shunt was recently described as allowing for both decreased risk of disease transmission and improved availability [22]. However, prions are still a risk in these corneas and the high cost is a major obstacle. These drawbacks have led some investigators to seek other solutions, such as placing the tube in a scleral tunnel [8] with or without Tenon advancement and a conjunctival–Tenon flap [7]. These descriptions, however, are all derived from anecdotal reports and the conventional surgical method continues to be the use of a patch graft in GDD surgeries.

The biologic material used as a patch graft in GDD surgeries has at least some role in preventing tube erosion [6], but discussions of these issues are sparse. One report [5] stated that no material was more prone to erosion or melting than another when comparing donor sclera, dura mater and pericardium. Those authors, however, reported a high incidence of donor patch graft thinning (22–26 %) for all 3 of those materials. A corneal patch graft may have some advantages over other covering materials. Its tissue strength and rigidity make it particularly suitable for tectonic support of the ocular wall, and it may be less prone to melting compared to other patch grafts [4, 22, 23]. Moreover, its translucency gives the patient’s eye a better cosmetic appearance [23], which is most relevant in cases where the GDD is placed in the inferior quadrants [22]. Migration, retraction and twisting are possible complications of the tube [24, 25], and they may be difficult to diagnose under an opaque patch graft, such as sclera or pericardium. The translucency of the cornea allows direct visualization of the underlying tube and greater facility in the diagnosis of possible complications [4]. It also facilitates laser suture lysis of the tying suture with non-valved tubes. We believe that the use of a partial thickness corneal graft offers satisfactory tectonic support and has some additional benefits over full thickness corneal grafts. The thin patch we use occupies little of the narrow space of the conjunctiva-sclera, thus minimizing the likelihood of the patch graft to erode the overlying conjunctiva. Indeed, none of our patients had that complication. The thin patch may also reduce the postoperative risk of dellen formation. Finally, the partial thickness corneal graft can be taken from a previous DSEK procedure or from a corneal graft that is unsuitable for optic corneal transplantation. This increases the availability of banked tissue without additional costs.

While a patch graft must be completely covered by the conjunctiva during the surgery, conjunctival laceration or button-hole formation may occur [10]. Simple suturing of the conjunctiva must then be carried out to ensure that the patch graft, tube and plate are entirely covered. This intra-operative complication occurred in one of our cases and was successfully dealt by prompt re-approximation of the conjunctival edges.

Limitations of this study include its retrospective nature, the relative small sample size and the lack of a control group with which to compare our results. Prospective randomized studies comparing partial thickness cornea to other patch graft materials are needed in order to determine which patch is less prone to conjunctival erosion and subsequent tube exposure. Cost considerations, the ideal thickness of the patch graft and its cosmetic appearance should also be taken into consideration. Since our analysis included only patients with follow-up of ≥12 months, complications occurring in patients with shorter follow-up were not reported, and this could have been a confounding factor. Although the mean postoperative follow-up time was more than 2 years, it was only 12 months in four patients. It is not clear whether a longer follow-up would reveal more cases of conjunctival dehiscence or corneal graft melting, but we consider it most probable that more of them would emerge over time. Some authors argued that these complications usually occur within the first 15-month postoperative period [5], while others held that these complications may occur even after 5 years [21]. Graft melting occurred 33 months after surgery in one of our patients, which suggests that patients who underwent GDD surgery should be examined regularly for years after surgery. The strict exclusion criteria that eliminated any cases of previous GDD surgery and patients with past and current ocular surface disease represent a strength of our study by allowing the evaluation of the partial thickness cornea as a patch graft without confounding factors that may influence conjunctival healing or erosion. Another strength is that all surgeries were done by one surgeon using the same technique, unlike most studies that evaluated the results and complications of GDDs placed by different surgeons using different techniques. Indeed, variations in techniques might affect tube exposure [6], making it difficult to draw any conclusions about the effectiveness of a specific patch graft in preventing the silicone tube exposure. We used a corneal patch that had an 8–9 mm diameter. The optimal size of a patch graft that will cover the tube completely from limbus to the plate has not yet been established, and this issue warrants further study.

## Conclusions

A partial thickness corneal graft is a useful patch graft material for covering a GDD. It is associated with low rates of conjunctival retraction, graft melting, tube exposure and need for additional surgery.

## Abbreviations

DSEK: descemet stripping endothelial keratoplasty
GDD: glaucoma drainage device
IOP: intraocular pressure
